# Upfront *DPYD* Genotyping and Toxicity Associated with Fluoropyrimidine-Based Concurrent Chemoradiotherapy for Oropharyngeal Carcinomas: A Work in Progress

**DOI:** 10.3390/curroncol29020045

**Published:** 2022-01-26

**Authors:** Antoine Desilets, William McCarvill, Francine Aubin, Houda Bahig, Olivier Ballivy, Danielle Charpentier, Édith Filion, Rahima Jamal, Louise Lambert, Phuc Felix Nguyen-Tan, Charles Vadnais, Xiaoduan Weng, Denis Soulières

**Affiliations:** Centre Hospitalier de l’Université de Montréal (CHUM), Montreal, QC H2X 3E4, Canada; william.mccarvill@umontreal.ca (W.M.); francine.aubin.chum@ssss.gouv.qc.ca (F.A.); houda.bahig.chum@ssss.gouv.qc.ca (H.B.); olivier.ballivy.chum@ssss.gouv.qc.ca (O.B.); danielle.charpentier.chum@ssss.gouv.qc.ca (D.C.); edith.filion.chum@ssss.gouv.qc.ca (É.F.); rahima.jamal.chum@ssss.gouv.qc.ca (R.J.); louise.lambert@gmail.com (L.L.); felix.nguyen.chum@ssss.gouv.qc.ca (P.F.N.-T.); charles.vadnais.chum@ssss.gouv.qc.ca (C.V.); xiaoduan.weng.chum@ssss.gouv.qc.ca (X.W.); denis.soulieres.chum@ssss.gouv.qc.ca (D.S.)

**Keywords:** *DPYD*, fluoropyrimidine, oropharyngeal cancer, chemoradiotherapy, head and neck cancer, pharmacovigilance, genotyping

## Abstract

**Simple Summary:**

The combination of carboplatin and 5-fluorouracil (5-FU) is effective when used concurrently with radiotherapy for locoregionally advanced oropharyngeal carcinomas. *DPYD* polymorphisms can be associated with an increased risk of severe toxicity to fluoropyrimidines. Upfront screening for the DPYD*2A allele has been available in the province of Québec, Canada, since March 2017. This study aimed to determine the effect of upfront genotyping on the incidence of grade ≥3 toxicities. We included 181 patients in the analysis. Extended screening for three supplemental at-risk *DPYD* variants was also retrospectively performed in August 2019. The DPYD*2A, c.2846A>T and c.1236G>A polymorphisms were associated with an increased risk of grade ≥3 toxicity to 5-FU. Upfront DPYD genotyping can thus identify patients in whom 5-FU-related toxicity should be avoided.

**Abstract:**

*Background*: 5-FU-based chemoradiotherapy (CRT) could be associated with severe treatment-related toxicities in patients harboring at-risk *DPYD* polymorphisms. *Methods*: The studied population included consecutive patients with locoregionally advanced oropharyngeal carcinoma treated with carboplatin and 5-FU-based CRT one year before and after the implementation of upfront DPYD*2A genotyping. We aimed to determine the effect of *DPYD* genotyping on grade ≥3 toxicities. *Results*: 181 patients were analyzed (87 patients before and 94 patients following DPYD*2A screening). Of the patients, 91% (*n* = 86) were prospectively genotyped for the DPYD*2A allele. Of those screened, 2% (*n* = 2/87) demonstrated a heterozygous DPYD*2A mutation. Extended genotyping of DPYD*2A-negative patients later allowed for the retrospective identification of six additional patients with alternative *DPYD* variants (two c.2846A>T and four c.1236G>A mutations). Grade ≥3 toxicities occurred in 71% of the patients before DPYD*2A screening versus 62% following upfront genotyping (*p* = 0.18). When retrospectively analyzing additional non-DPYD*2A variants, the relative risks for mucositis (RR 2.36 [1.39–2.13], *p* = 0.0063), dysphagia (RR 2.89 [1.20–5.11], *p* = 0.019), and aspiration pneumonia (RR 13 [2.42–61.5)], *p* = 0.00065) were all significantly increased. *Conclusion*: The DPYD*2A, c.2846A>T, and c.1236G>A polymorphisms are associated with an increased risk of grade ≥3 toxicity to 5-FU. Upfront *DPYD* genotyping can identify patients in whom 5-FU-related toxicity should be avoided.

## 1. Introduction

Fluoropyrimidines, such as 5-fluorouracil (5-FU) and prodrug capecitabine, are pyrimidine analogs that interfere with DNA and RNA synthesis, which have shown activity in the treatment of gastrointestinal tract malignancies, as well as head and neck squamous cell carcinoma (HNSCC) [1,2,3,4]. Severe and potentially life-threatening toxicities (grade 3 or 4 according to the Common Terminology Criteria for Adverse Events (CTCAE) version 5 [5]) occurring early after the start of therapy affect approximately 5–15% [6,7] of the population treated with fluoropyrimidines. Chemotherapy must usually be discontinued upon such adverse events and deleterious impacts on quality of life and prognosis can ensue, with frequent hospitalizations and incurring healthcare costs. Efforts have thus been made to preemptively identify patients at increased risk of severe toxicity following standard dosing regimens of 5-FU or capecitabine.

Fluoropyrimidines are metabolized in the liver in a multi-step process. The initial and rate-limiting enzyme in pyrimidine catabolism is dihydropyrimidine dehydrogenase (DPD), which inactivates more than 80% of 5-FU [8,9]. This enzyme is encoded by the *DPYD* gene [10]. Genetic *DPYD* polymorphisms can result in partial or complete DPD deficiency [11]. According to previous studies, 3–5% of Caucasians are thought to be poor metabolizers. Multiple *DPYD* variant alleles have been described, with DPYD*2A (IVS14+1G>A) being the most studied, affecting 1–2% of Caucasian populations in the heterozygous state [12,13,14]. The c.2846A>T [15], c.1679T>G [16], and c.1236G>A [16] mutations are also associated with decreased DPD activity. In Asian populations, the rs59086055 and rs186169810 haplotypes have additionally been linked to poor 5-FU catabolism [17]. Fluoropyrimidine toxicity has been associated with reduced DPD activity and DPYD*2A polymorphism, based on numerous reports [18,19,20,21,22,23,24], including two recent meta analyses [16,25].

5-FU has radiation-sensitizing properties [26]. In locally advanced HNSCC, concurrent chemoradiotherapy (CRT) improves locoregional control and survival compared to radiotherapy alone [27]. Moreover, when used as a definitive treatment, this strategy has the benefit of being organ-sparing and is now considered the standard of care. Cisplatin is often employed, alone or in combination chemotherapy regimens, for HNSCC in North America [27,28]. The carboplatin and 5-FU regimen represents an alternative option and has been shown to be effective when used concurrently with radiotherapy for locoregionally advanced oropharyngeal squamous cell carcinomas (OPSCCs) [29,30]. Moreover, a meta-analysis by Browman et al. demonstrated that combination chemotherapy is superior to single-agent chemotherapy in combination with radiation therapy for locally advanced diseases [31]. A local study previously demonstrated comparable overall survival (OS) data for OPSCC patients treated with carboplatin and 5-FU (when compared to cisplatin), with rates of grade 3 neutropenia favoring the combination regimen [30]. When 5-FU is prescribed in this setting, doses are two to three times lower than those used for gastrointestinal neoplasms (mFOLFOX6 [32]). A substantially increased risk of toxicity persists due to the synergistic effect of radiation therapy [33]. Despite limited evidence, the addition of carboplatin–paclitaxel to radiotherapy, which is mainly used in the United States, also represents an option for locally advanced HNSCC. The National Comprehensive Cancer Network (NCCN) guidelines include the combination as an alternative CRT regimen in such a setting, although the recommendation currently stands as category 2B. As such, no phase III clinical trial has yet concluded on the benefits of such an approach. In addition, the toxicity profile associated with paclitaxel-based CRT remains largely unreported for head and neck cancer. In view of such absence of randomized trials for paclitaxel–carboplatin, CRT regimens studied in phase III trials represent more evidence-based options for HNSCC patients, especially in the context of pharmacogenomic strategies allowing for chemotherapy modulation, as in the case of the carboplatin and 5-FU combination, which is the object of the current analysis.

Upfront genotyping for the DPYD*2A polymorphism is available in the province of Quebec (Canada) and covered by the government [34]. The test employs real-time polymerase chain reaction (PCR) to detect DPYD*2A allele polymorphisms. RT-PCR, sometimes referred to as quantitative PCR (qPCR), is a molecular biology technique monitoring the amplification of targeted nucleic acid sequences using specific DNA probes. Such probes consist of fluorescently-labeled oligonucleotides, which hybridize to their complementary sequences (i.e., *DPYD* genetic polymorphisms), thus allowing for their detection. RT-PCR has been performed at the Centre Hospitalier de l’Université de Montréal (CHUM) since March 2017 and is a verified and validated assay according to the *Bureau de Normalisation du Québec* guidelines.

Genomic DNA was extracted from peripheral blood mononuclear cells (PBMCs) using the Qiasymphony system (Qiagen Inc., Germantown, MD, USA). RT-PCR screening for variant DPYD*2A alleles (c.1905+1G>A, IVS14+1G>A, and rs3918290), c.2846A>T (rs7376798 and D949V), c.1679T>G (rs55886062, DPYD*13, and I560S), and c.1236G>A (rs56038477 and E412E) was performed through TaqMan primers and probes (Thermo Fisher Scientific, Waltham, MA, USA). Positive controls (heterozygote variant DNA) were provided by Dr Jan Schellens from the Netherlands Cancer Institute, Utrecht University, the Netherlands [35]. Our institutional RT-PCR assays were validated internally and externally and approved for clinical use by the *Institut National d’Excellence en Santé et Services Sociaux* (INESSS). For patients identified as heterozygous for the DPYD*2A allele and for whom treatment with a dihydropyrimidine is mandated, an initial 5-FU dose reduction of at least 50% is normally suggested [36]. Further dose adjustments are performed based on tolerance.

In their analysis published in 2016, Deenen et al. studied the cost-effectiveness of upfront DPYD*2A screening prior to initiating fluoropyrimidines. In the studied population, 1.1% (*n* = 22) of patients were found to be heterozygous for the DPYD*2A allele [37]. The authors ultimately demonstrated a decrease in treatment-related toxicities, as well as modest healthcare cost reductions, upon upfront genotyping. Most of the patients included in the study, however, presented diagnoses of colorectal or breast cancer, with only one HNSCC patient identified with the DPYD*2A mutation. In this regard, little data are available concerning the potential clinical benefits of *DPYD* screening in the HNSCC population, especially when considering the added toxicities associated with concurrent CRT.

We hypothesized that upfront DPYD*2A genotyping could improve patient safety by decreasing toxicity rates and hospitalizations on a populational level, with ensuing decreased healthcare expenditures. We also hypothesized that extended *DPYD* genotyping could identify additional at-risk allele carriers and further decrease treatment-attributable adverse events. More specifically, we aimed to evaluate the association between *DPYD* mutational status and incidence of 5-FU-related adverse events, such as mucositis, as well as the associated consequences of dysphagia, pharyngolaryngeal pain, and aspiration pneumonia. To this effect, the modulation of 5-FU toxicity through systematic *DPYD* screening could improve patient outcomes and represent a safe alternative to cisplatin-based CRT regimens for OPSCC.

## 2. Materials and Methods

### 2.1. Study Design

This was a single-center observational cohort study conducted at the CHUM between March 2017 and April 2018, with both retrospective and prospective components. All clinical data were extracted from chart review. Detailed safety data were available for all patients in the study from weekly radiation oncology reports and medical oncology assessments before every chemotherapy cycle. Adverse events were characterized according to their highest reported grade through longitudinal follow-up.

Considering the low prevalence of the DPYD*2A allele and since randomization would be considered unethical, patients from the upfront genotyping prospective cohort were compared to a local cohort of consecutive OPSCC patients retrospectively identified from the year prior to DPYD*2A screening implementation (February 2016 to February 2017). Patients’ factors thought to harbor a prognostic impact, including disease stage, smoking status, and tumor HPV association (as assessed through p16 antigenic identification [38]), were also collected for both groups.

In June 2018, extended screening for three supplemental at-risk *DPYD* variants (c.2846A>T, c.1679T>G, and c.1236G>A mutations) [15,16] was introduced at the CHUM after approval from provincial regulatory agencies [34]. Patients with retrospectively identified supplemental at-risk *DPYD* variants were treated with carboplatin and 5-FU-based chemoradiation considering the absence of DPYD*2A mutation on prospective RT-PCR screening. Re-analyzing such patients’ stored DNA samples, *DPYD* polymorphisms could be correlated with treatment-related adverse events.

**Figure 1:**
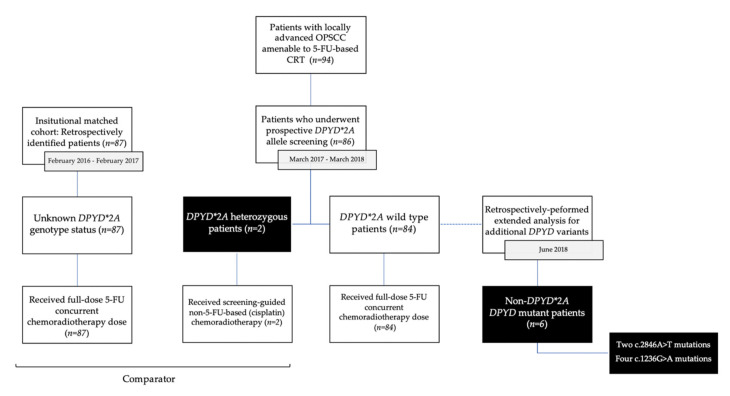
CONSORT Diagram. This diagram illustrates patients’ flow following initial *DPYD* screening and subsequent treatment according to the presence of the DPYD*2A polymorphism. Toxicities were compared between the screening cohort and a local cohort consisting of retrospectively identified patients treated with carboplatin and 5-FU-based chemoradiation. Screening for additional at-risk *DPYD* variants was later retrospectively performed in June 2018.

The primary endpoint was the incidence of severe mucositis (CTCAE grades 3 to 5). Secondary endpoints included overall grade ≥3 toxicities, as well as the incidence of dysphagia, pharyngo-laryngeal pain, aspiration pneumonia, hematological toxicities, hospitalization, and enteral feeding requirements. The study was approved by the institutional ethics committee (REB number: 18.235-MJB, 2019-7675). The CONSORT diagram is presented in Figure 1.

### 2.2. Patient Population

The study population consisted of patients diagnosed with locoregionally advanced OPSCC for which concurrent chemotherapy with 5-FU was considered as definitive therapy (stage III, IVA and IVB OPSCC according to the seventh edition [39] of the American Joint Committee on Cancer [AJCC] staging manual). The use of the seventh edition’s staging system was preferred for homogeneity purposes, since part of the cohort was diagnosed before 2017. All patients received both their systemic treatment and radiation therapy at the CHUM, where longitudinal follow-up was performed.

### 2.3. Treatment

Patients were prospectively genotyped for the DPYD*2A variant through germline DNA RT-PCR analysis. *DPYD* wild-type (WT), as well as unscreened patients, were treated with three cycles of a four-day regimen containing carboplatin (70 mg/m^2^ on day 1) and 5-FU (600 mg/m^2^ per day by continuous infusion). Heterozygous (or homozygous) DPYD*2A allele carriers were alternatively treated with three cycles of high-dose (100 mg/m^2^) cisplatin-based concurrent CRT. Both groups received radiotherapy to the primary tumor and involved lymph nodes, either through standard fractionation at a dose of 70 Gy in 35 daily fractions (including concomitant boost) or through intensity-modulated radiation therapy (IMRT) at a dose of 70 Gy in 33 fractions. Since it was obtained retrospectively, extended *DPYD* genotyping had no impact on the initial treatment assignment, and differential analysis of adverse events could thus be performed comparing the non-DPYD*2A mutant and *DPYD*-WT populations.

### 2.4. Statistical Analysis

In the retrospective analysis (i.e., patients assessed for non-DPYD*2A mutations and their correlation to 5-FU-mediated toxicities), the clinical endpoints were hierarchically examined according to *DPYD* mutational status. The study was designed to demonstrate a 30% decrease in severe mucositis events with a statistical power of 90%. If the primary endpoint is demonstrated, the overall incidence of grade ≥3 chemoradiotherapy-related complications would then be analyzed. Similarly, upon a 30% decrease in overall toxicity, specific adverse events attributable to 5-FU were then examined. This hierarchical statistical plan is designed to prevent adjustment of the power for multiple comparisons.

Baseline patient and tumor characteristics were compared using Fisher’s exact test for binary outcomes or using the chi-squared test otherwise. All data were analyzed according to a per-protocol analysis. Since this study is essentially retrospective in nature, all reported statistical analyses are descriptive in nature. *DPYD* genotypes were considered binary variables for the individual analyses (DPYD*2A vs. non-DPYD*2A for the prospective analysis, *DPYD* WT vs. non-DPYD*2A mutants for the retrospective analysis). Relative risk (RR) was defined as the ratio of the toxicity risk in patients positive for selected non-DPYD*2A mutations to that of patients wild-type for extended *DPYD* mutational screening. Using the Koopman asymptotic score, 95% confidence intervals were computed. The associations between adverse events and genotype status were tested according to the chi-squared test. Statistical significance was set at a *p*-value of <0.05 and all tests were two-sided. Statistical analyses were conducted using GraphPad Prism.

## 3. Results

### 3.1. Patient Characteristics

Between March 2017 and April 2018, 94 consecutive patients with OPSCC amenable to definitive concurrent CRT for locally advanced disease were identified. Of those, 91% (*n* = 86) were prospectively genotyped for the DPYD*2A allele in order to guide clinical decision-making in terms of the chemotherapy regimen. In the comparator group, 87 consecutive patients were retrospectively identified with OPSCC between February 2016 and February 2017. The baseline demographics and patient characteristics for both groups are presented in Table 1. There were no differences between the screened and the unscreened subgroups in terms of baseline characteristics.

While most patients included in the study were active or past smokers, 89% and 95% of patients in both cohorts presented oropharyngeal tumors associated with HPV infection, respectively, as defined by p16 antigenic overexpression on immunohistochemistry. The majority of patients presented stage IVA or IVB disease, defined as T4 and/or N2–N3 disease [39].

Induction chemotherapy for locoregionally advanced tumors was administered to 21% and 17% of patients in each group, respectively. The majority of patients completed either two or three cycles of 5-FU in combination with carboplatin. Two patients in each group prematurely stopped chemotherapy after one cycle, either due to life-threatening toxicities (coronary vasospasm, and severe mucositis), performance status deterioration, or following patients’ withdrawal of consent. All patients completed their radiation therapy treatments.

Of the 86 patients tested for the DPYD*2A polymorphism, two cases of heterozygous mutations were identified, both in Caucasian males (ages 56 and 61). These two patients had no cardiovascular risk factors and were alternatively treated with high-dose cisplatin chemotherapy in the course of their concurrent radiotherapy regimen. Six additional heterozygous mutations were later retrospectively identified upon extended *DPYD* genotyping. Two of those patients harbored the c.2846A>T gene variant and four patients presented the c.1236G>A variant. No homozygous mutation was identified. Table 2 summarizes the *DPYD* genotyping data.

### 3.2. Safety Data

Safety data were available for all patients in the study. Local symptoms characterized by mucositis, dysphagia, or radiation-induced dermatitis were the most prevalent toxicities. Grade ≥3 mucositis events occurred in 54% of patients (*n* = 47) prior to DPYD*2A screening implementation, versus in 47% (*n* = 40) following genotyping (*p* = 0.32). Severe (CTCAE grade ≥3) toxicities occurred in 71% and 65% (*n* = 61/94) of patients in the pre- and post-implementation periods, respectively. Neutropenic episodes were observed in 8–10% of patients, including cases of febrile neutropenia in three patients preceding and six patients following systematic *DPYD* screening. Rare toxicities were also recorded following the second or third cycle of 5-FU-based concurrent CRT. Those included cases of catastrophic intravascular hemolysis requiring plasmapheresis and blood product transfusion, severe acute kidney injury on hemodialysis support and two cases of radiation-induced osteonecrosis. No treatment-related mortality was described in the total population.

Medical interventions for severe adverse events included feeding tube installation, antibiotics administration, blood products transfusion, and topical application of silver sulfadiazine (Flamazine^®^ [Smith & Nephew], London, United Kingdom or Mepilex^®^ [Mölnlycke], Gothenburg, Sweden) for radiation-induced dermatitis. One patient from the pre-screening cohort developed aspiration pneumonia from mucositis and severe dysphagia, eventually requiring mechanical ventilation in the intensive care unit. Globally, 29% of the patients were hospitalized prior to DPYD*2A genotyping, versus 22% (*n* = 21/94) in the post-screening period.

In the two patients prospectively identified with heterozygous DPYD*2A mutations and treated with cisplatin-based CRT, no grade ≥3 toxicities or hospitalizations were recorded. Patients retrospectively found to harbor heterozygous c.2846A>T and c.1236G>A mutations demonstrated high rates of severe adverse events, including grade 3 mucositis in all cases, dysphagia in 66%, and aspiration pneumonia in 33%. In this same subgroup, 33% were hospitalized and 50% required enteral feeding.

Considering the low prevalence of *DPYD* polymorphisms, no statistically significant reduction in terms of toxicities or hospitalizations was recorded following DPYD*2A genotyping implementation for OPSCC. Subgroup analysis according to retrospective and extended *DPYD* mutational status characterization, however, demonstrated a 58% increase in grade ≥3 mucositis for patients later shown to be carriers of selected mutant alleles. The relative risk (RR) of mucositis was 2.36 times (95% CI 1.38–2.13) higher in the non-DPYD*2A-mutant patients (*p* = 0.0063). Following such a finding and taking into account our initial statistical plan, overall grade ≥3 toxicities were later assessed. To this extent, *DPYD* wild-type status was found to be associated with a 41% decrease in overall severe adverse events, with a RR of 1.70 (1.01–2.09) (*p* = 0.046). Hence, all secondary endpoints were analyzed and compared between the two groups and are hereby presented in Table 3. The treatment interventions in each arm are presented in Table 4.

The Supplementary Materials provide a comparative analysis of toxicities reported in the pre- and post-DPYD*2A screening periods (Supplementary Tables S1 and S2), as well as individual descriptive data for all consecutive patients identified with *DPYD* mutations (Table S3). Of note, all six patients with retrospectively identified at-risk *DPYD* polymorphisms and who were treated with carboplatin and 5-FU-based CRT were still in clinical remission as of the latest medical follow-up (2021).

## 4. Discussion

To the best of our knowledge, this analysis represents the first application of *DPYD* genotyping in a population of locally advanced OPSCC patients amenable to 5-FU-based CRT. The study indicated that upfront screening for *DPYD* mutations could decrease the risk of severe toxicities associated with 5-FU-based CRT. Genotyping implementation was feasible in clinical practice, and was quickly adopted by medical oncologists. Even though the analysis was underpowered in order to attain statistical significance in the prospectively screened cohort, subgroup analysis upon extended genotyping demonstrated a trend toward increased risk of severe mucositis, dysphagia, and aspiration pneumonia in the non-DPYD*2A mutant subpopulation. As institutional screening for additional variants was only implemented in June 2017, severe toxicities in such an at-risk population could have been prevented upon prospective testing. When compared to *DPYD*-WT patients, patients harboring c.2846A>T or c.1236G>A mutations could also present higher hospitalization rates. Of note, the increased median hospitalization duration described in the *DPYD*-WT population was mainly driven by a few cases of aspiration pneumonia requiring a prolonged hospital stay and ventilatory support. Patients with retrospectively identified non-DPYD*2A polymorphisms also appeared to require more frequent antibiotics use and feeding tube requirements. As primary and secondary endpoints were inter-related from a causality perspective (i.e., mucositis being the main 5-FU chemoradiotherapy-attributable adverse event), multivariate analysis was deemed non-contributory and thus omitted from our statistical plan.

Deenen et al. demonstrated that 5-FU dose reductions across a wide range of tumor histologies were associated with a decreased risk of severe toxicities [37]. Dose individualization was not thought to affect treatment efficacy based on pharmacokinetic analyses describing similar 5-FU drug exposure following dose reductions in DPYD*2A mutant patients. In the current study, patients identified with DPYD*2A mutations were alternatively treated with cisplatin-based CRT. As previously mentioned, the institutional decision to offer carboplatin and 5-FU-based CRT as the standard-of-care for locally advanced OPSCC is based on a local study evaluating the toxicity profiles of different chemotherapy regimens in combination with concurrent radiation therapy. When compared to cisplatin, Barkati et al. determined that the 5-FU doublet is associated with a decreased risk of neutropenia [30], without impacting treatment responses. No difference in OS when administering two versus three cycles of carboplatin–5-FU was recognized upon survival data analysis of patients who failed to complete their final cycle due to adverse events. However, the study was underpowered in order to demonstrate equivalence between such subgroups. In our study, no grade ≥3 toxicities were reported in patients treated with cisplatin. While this sample is not representative of the full scope of cisplatin-associated complications, it is also important to note that both DPYD*2A carriers presented a favorable comorbidity profile and good anticipated treatment tolerance. As upfront DPYD*2A genotyping was unblinded to the investigators, it is also possible that such a low rate of toxicities may be partially attributable to unintended closer surveillance in this at-risk population. Consequently, a reduction in 5-FU-related toxicity with the carbo-5-FU regimen could lead to significant reductions in chemo-radiation toxicity compared to the cisplatin-based regimen.

Following DPYD*2A screening implementation in March 2017, 2% (*n* = 2/86) of patients were identified with heterozygous mutations. This prevalence is similar to the reported rates in the literature, with 1% of patients being carriers of the DPYD*2A allele in Deenen et al.’s analysis [37]. In the present study, both patients identified were Caucasian males with no prior cancer history or exposure to fluoropyrimidines. Upon application of an extended *DPYD* genotyping program in June 2018, six additional patients were retrospectively identified with non-DPYD*2A mutations, which represented 2% of the population for the c.2846A>T variant allele (*n* = 2/86) and 5% (*n* = 4/86) for the c.1236G>A mutation. Non-DPYD*2A mutations in this study were overrepresented compared to published data in the literature. In fact, historic databases describe a prevalence of 1.5-–2% for the c.1236G>A polymorphism and of 0.1–0.5% for c.2846A>T [40]. As the DPYD*2A allele is associated with a reduction in DPD enzyme activity of approximately 50%, other variants (such as c.2846A>T and c.1236G>A) show an average decrease of 25% in terms of fluoropyrimidine catabolism activity [41]. In historical cohorts of patients treated with 5-FU without dose individualization, DPYD*2A mutations were associated with a 2.85 relative risk (RR) of severe toxicities, a RR of 1.52 for the c.1236G>A variant and a RR of 3.02 for c.2846A>T [16].

In this study, only 91% (*n* = 86) of the population amenable to *DPYD* genotyping was prospectively tested for the DPYD*2A polymorphism prior to CRT initiation. Such suboptimal compliance can be attributed to the initial adaptation process upon clinical implementation of a new pharmacovigilance strategy. Auditing of recent patients’ data indicates systematic testing of all cancer cases for which fluoropyrimidine therapy is indicated, as *DPYD* testing is now included in the baseline laboratory work of all applicable chemotherapy protocols. A higher detection yield is therefore expected in the future. Finally, a recent Quebec-wide study conducted by Jolivet et al. analyzed the effect of DPYD*2A genotyping on treatment administration schedules [42]. The authors concluded that results from DPYD*2A screening were available in an average of six days across the province and that testing was not associated with delays in terms of treatment initiation according to 99% of the polled physicians.

When combined with *DPYD* genotyping, carboplatin and 5-FU-based CRT thus seem to be well tolerated in patients with locally advanced OPSCC. Data previously published in the literature [26,27] also demonstrated the non-inferiority of this regimen when compared to cisplatin-based CRT. Modulation of 5-FU toxicity through extended *DPYD* screening could therefore represent an efficacious alternative, if not a safer option, compared to cisplatin- or carboplatin–paclitaxel-based CRT. In view of the paucity of data in the literature currently justifying a chemotherapy regimen other than cisplatin or carboplatin and 5-FU for oropharyngeal cancer, we argue that the carboplatin and 5-FU combination following *DPYD* screening could represent a better-suited option for this specific population. The extent to which *DPYD* screening affects patient outcomes, not only in terms of toxicity, but also in terms of efficacy, however, needs to be further studied. Ideally, the demonstration of this study would be validated in additional patient cohorts. However, ethical concerns would be raised by denying *DPYD* testing, which would hamper the possibility of conducting such a research initiative, as extended screening is now universally available and recommended with the accepted potential of preventing *DPYD* mutation-associated fluoropyrimidine toxicities. Even though the study presented here does not have the statistical strength of a prospective study, we propose that it informs on the likelihood of developing important complications if fluoropyrimidine metabolism is not investigated before a treatment with these agents and radiation therapy in locally advanced SCC.

## 5. Conclusions

The DPYD*2A, c.2846A>T, and c.1236G>A polymorphisms are associated with an increased risk of severe toxicity to 5-FU in locally advanced OPSCC patients, as well as higher hospitalization rates when compared to *DPYD*-WT patients. Upfront *DPYD* genotyping can identify patients in which fluoropyrimidine-related toxicity could be avoided and its institutional implementation could be promising from a pharmacovigilance perspective. Cisplatin-based CRT represents a viable option for DPYD*2A carriers. In a primarily Caucasian population, a total of 9% of patients presented at-risk *DPYD* polymorphisms. A prevalence rate of 2% was described for the DPYD*2A allele, of 2% for the c.2846A>T variant and of 5% for the c.1236G>A mutation.

In the era of personalized medicine, the discovery of patient characteristics should lead to informed decisions on the balance between benefit and harm associated with treatment. It is therefore likely that previously uninvestigated germline single nucleotide polymorphisms (SNPs) will question the safety of proposed therapies. Much like uncontrolled next generation sequencing (NGS) analysis of a tumor reveals unforeseen mutations of unknown significance and possible treatment options, mutations in genes known to be involved in drug metabolism need to be considered.

The data presented are part of a work in progress to adequately identify patients at risk of significant adverse events from chemotherapy, specifically fluoropyrimidines. In fact, other genotypes have been identified in non-Caucasians, and these, as well as the four genotypes reported here, need to be tested and validated in a larger cohort of patients

## Figures and Tables

**Table 1:** Baseline characteristics.

Characteristics	Pre-DPYD*2A Genotyping Patients (*n* = 87)	Post-DPYD*2A Genotyping Patients (*n* = 94)	*p*
Age at diagnosis (years)			
Median	62	60	-
<65	60% (*n* = 52)	63% (*n* = 66)	0.16
≥65	40% (*n* = 35)	37% (*n* = 38)	
Sex			
Male	71% (*n* = 62)	80% (*n* = 75)	0.23
Female	29% (*n* = 25)	20% (*n* = 19)	
Smoking history			
Yes	80% (*n* = 70)	78% (*n* = 73)	0.47
Number of pack-years (median)	34.5	30	
Active	20% (*n* = 17)	13% (*n* = 12)	
Never	20% (*n* = 17)	22% (*n* = 21)	
p16 antigen overexpression ^†^			
Patients screened for p16 status	74% (*n* = 64)	89% (*n* = 84)	-
p16-positive patients	89% (*n* = 57)	95% (*n* = 80)	0.21
p16-negative patients	11% (*n* = 7)	5% (*n* = 4)	
Herpes simplex virus (HSV) status ^‡^			
Patients screened for HSV	54% (*n* = 47)	70% (*n* = 66)	0.58
HSV-1- and/or HSV-2-positive	89% (*n* = 42)	85% (*n* = 56)	
Staging (AJCC 7th edition ^§^)			
III	10% (*n* = 9)	7% (*n* = 7)	0.32
IVA	69% (*n* = 60)	79% (*n* = 74)	
IVB	21% (*n* = 18)	14% (*n* = 13)	
TNM descriptors			
Primary tumor			
Tx	11% (*n* = 10)	4% (*n* = 4)	0.17
T1	9% (*n* = 8)	19% (*n* = 18)	
T2	32% (*n* = 28)	33% (*n* = 31)	
T3	24% (*n* = 21)	23% (*n* = 22)	
T4	23% (*n* = 20)	20% (*n* = 19)	
Lymph node status			
N0	7% (*n* = 6)	2% (*n* = 2)	0.38
N1	8% (*n* = 7)	10% (*n* = 9)	
N2	70% (*n* = 61)	77% (*n* = 72)	
N3	15% (*n* = 13)	12% (*n* = 11)	
First-line chemotherapy			
Induction chemotherapy			0.68
Docetaxel, cisplatin and 5-FU (TCF)	6% (*n* = 5)	3% (*n* = 3)	
Docetaxel and cisplatin	15% (*n* = 13)	14% (*n* = 13)	
Carboplatin and 5-FU	79% (*n* = 69)	83% (*n* = 78)	
Number of 5-FU cycles completed (for non-mutated DPYD*2A patients)			
1	2% (*n* = 2)	2% (*n* = 2)	0.88
2	52% (*n* = 45)	55% (*n* = 51)	
3	46% (*n* = 40)	42% (*n* = 39)	

^†^ Based on immunohistochemistry. ^‡^ Herpes simplex virus (HSV) serological screening. ^§^ American Joint Committee on Cancer 7th Edition (prior to updated HPV-status dichotomized staging, for homogeneity purposes)

**Table 2:** *DPYD* genotyping ^†^.

Characteristics	Genotyped Patients
First analysis: Prospective DPYD*2A genotyping (*n* = 94)	
Patients initially screened for DPYD*2A	91% (*n* = 86)
DPYD*2A allele carriers	
Heterozygote	2% (*n* = 2)
Homozygote	0% (*n* = 0)
Second analysis: Retrospective extended *DPYD* genotyping ^‡^ (*n* = 86)	
Patients undergoing extended *DPYD* screening	100% (*n* = 86)
Non-DPYD*2A mutant alleles	
c.2846A>T	2% (*n* = 2)
c.1679T>G	0% (*n* = 0)
c.1236G>A	5% (*n* = 4)
Combined analysis (*n* = 86)	
Patients harboring *any* clinically significant *DPYD* mutant allele ^§^	9% (*n* = 8)

^†^ Including retrospective extended mutant alleles identification. ^‡^ Requiring an initial DPYD*2A PCR DNA specimen. ^§^ Previously described genetic polymorphisms associated with DPD enzymatic deficiency.

**Table 3:** Patient clinical and laboratory severe toxicities (grade ≥3) ^†^.

Adverse Events	Post-DPYD*2A Genotyping Patients (*n* = 86)			*p*
	*DPYD*-WT Patients (*n* = 78)	Non-DPYD*2A-Mutant Patients (*n* = 6) ^‡^	RR (95% CI)	
Patients with available longitudinal toxicity data	100% (*n* = 78)	100% (*n* = 6)	N/A	N/A
*Mucositis*	42% (*n* = 33)	100% (*n* = 6)	2.36 (1.39–2.13)	0.0063
Overall grade ≥3 toxicity	59% (*n* = 46)	100% (*n* = 6)	1.70 (1.01–2.09)	0.046
Other clinical toxicity (secondary endpoints)				
*Dysphagia*	23% (*n* = 18)	66% (*n* = 4)	2.89 (1.20–5.11)	0.019
*Pharyngolaryngeal pain*	12% (*n* = 9)	50% (*n* = 3)	4.33 (1.41–10.2)	0.0095
*Aspiration pneumonia*	3% (*n* = 2)	33% (*n* = 2)	13 (2.42–61.5)	0.00065
*Radiation-induced dermatitis*	14% (*n* = 11)	0% (*n* = 0)	0 (0–3.05)	0.32
Xerostomia	1% (*n* = 1)	17% (*n* = 1)	13 (1.39–110)	0.017
Cellulitis	1% (*n* = 1)	17 (*n* = 1)	13 (1.39–110)	0.017
Laboratory toxicities				
*Neutropenia*	9% (*n* = 7)	17 (*n* = 1)	1.86 (0.31–8.23)	0.54
Thrombocytopenia	4% (*n* = 3)	17% (*n* = 1)	4.33 (0.64–24.0)	0.16
Anemia	3% (*n* = 2)	0% (*n* = 0)	0 (0–20.5)	0.69

In *italic type*: Patients’ clinical and laboratory adverse events most likely related to 5-FU administration. ^†^ Severe toxicities reported in more than 1% of patients. ^‡^ Retrospectively performed genotyping (keeping in mind that upfront DPYD*2A screening allowed investigators to omit 5-Fexposure for patients prospectively identified with the DPYD*2A polymorphism).

**Table 4:** Medical interventions indicated for previously reported toxicities.

Adverse Events	Post-DPYD*2A Genotyping Patients (*n* = 86)			*p*
	*DPYD*-WT Patients (*n* = 78)	Non-DPYD*2A-Mutant Patients (*n* = 6) ^†^	RR (95% CI)	
Patients with available longitudinal toxicity data	100% (*n* = 78)	100% (*n* = 6)	N/A	N/A
Patients requiring hospitalization				
≥1 hospitalization	23% (*n* = 18)	33% (*n* = 2)	0.69 (0.28–2.50)	0.57
Median duration (days)	7	4.5	N/A	N/A
Patients requiring special treatment				
Enteral feeding	32% (*n* = 25)	50% (*n* = 3)	1.56 (0.56–2.95)	0.37
Antibiotics	13% (*n* = 10)	33% (*n* = 2)	2.6 (0.69–7.2)	0.17

^†^ Retrospectively performed genotyping (keeping in mind that upfront DPYD*2A screening allowed investigators to omit 5-FU exposure for patients; prospectively identified with the DPYD*2A polymorphism).

## Data Availability

Data supporting the reported results are stored on a secure server at the CHUM and are accessible through the corresponding author.

## Supplementary Materials

The following are available online at https://www.mdpi.com/article/10.3390/curroncol29020045/s1, Table S1: Patient clinical and laboratory severe toxicities (grade ≥ 3); Table S2: Medical interventions indicated for previously reported toxicities; Table S3: Individual descriptive data for consecutive patients identified with *DPYD* polymorphisms
