# Supplementary material for: Upfront DPYD Genotyping and Toxicity Associated with Fluoropyrimidine-Based Concurrent Chemoradiotherapy for Oropharyngeal Carcinomas: A Work in Progress
Source: Curr Oncol. 2022 Jan 26;29(2):497–509. doi: 10.3390/curroncol29020045 (PMC8870563; doi:10.3390/curroncol29020045)
Supplement: Supplementary file 1 [file curroncol-29-00045-s001.zip › curroncol-1477847-supplementary.pdf]

Current Oncology

Supplementary Materials

Table S1. Patient Clinical and Laboratory Severe Toxicities (grade ≥ 3) †

**Adverse events**	**Pre-*DPYD*2A* genotyping patients (*n* = 87)**	**Post-*DPYD*2A* genotyping patients (*n* = 86)**	***p***
Patients with available longitudinal toxicity data	100% (*n* = 87)	100% (*n* = 86)	N/A
Mucositis	54% (*n* = 47)	47% (*n* = 40)	0.32
Overall severe toxicity	71% (*n* = 62)	62% (*n* = 53)	0.18
Clinical toxicity			
Dysphagia	39% (*n* = 34)	26% (*n* = 22)	0.058
Pharyngolaryngeal pain	9% (*n* = 8)	14% (*n* = 12)	0.32
Pneumonia (including aspiration events)	1% (*n* = 1)	5% (*n* = 4)	0.17
Radiation-induced dermatitis	15% (*n* = 13)	13% (*n* = 11)	0.68
Xerostomia	1% (*n* = 1)	2% (*n* = 2)	0.55
Cellulitis	1% (*n* = 1)	2% (*n* = 2)	0.55
Laboratory toxicities			
Neutropenia	8% (*n* = 7)	9% (*n* = 8)	0.77
Thrombocytopenia	5% (*n* = 5)	5% (*n* = 4)	0.75
Anemia	4% (*n* = 4)	2% (*n* = 2)	0.41

In *italic type*: patient clinical and laboratory adverse events most likely related to 5-FU administration. † Severe toxicities reported in more than 1% of patients

Table S2. Medical Interventions Indicated for Previously Reported Toxicities.

**Adverse events**	**Pre-*DPYD*2A* genotyping patients (*n* = 87)**	**Post-*DPYD*2A* genotyping patients (*n* = 86)**	***p***
Patients with available longitudinal toxicity data	100% (*n* = 87)	100% (*n* = 86)	N/A
Patients requiring hospitalization			
≥ 1 hospitalization	29% (*n* = 25)	23% (*n* = 20)	0.41

Median duration (days)	3	6	N/A
Patients requiring special treatment			
Enteral feeding	41% (n = 36)	33% (n = 28)	0.23
Antibiotics	13% (n = 11)	14% (n = 12)	0.80
≥ 1 blood product transfusion	3% (n = 2)	0% (n = 0)	0.16

Table S3. Individual Descriptive Data for Consecutive Patients Identified with DPYD Polymorphisms.

**Patient ID**	**1**	**2**	**3**	**4**	**5**	**6**	**7**	**8**
DPYD polymorphism								
*DPYD*2A* mutation	**Yes**	**Yes**	No	No	No	No	No	No
Other *DPYD* variants	No	No	**c.2846A>T**	**c.2846A>T**	**c.1236G>A**	**c.1236G>A**	**c.1236G>A**	**c.1236G>A**
Patient baseline characteristics								
Age (years)	56	61	73	59	40	52	63	50
Sex	M	M	M	M	M	F	F	M
Smoking status	Past	Never	Past	Past	Past	Past	Past	Never
HSV status	HSV1+	HSV1+	HSV1+	Unknown	HSV1+	Unknown	Unknown	Unknown
p16 antigen status	?	HPV+	HPV+	HPV+	HPV+	HPV+	HPV+	HPV+
Staging (Tx Nx M0)	T4a N1 (IVA)	T2 N2c (IVA)	T1 N3 (IVB)	T3 N2b (IVA)	T2 N2b (IVA)	T2 N2b (IVA)	T2 N2c (IVA)	T1 N2b (IVA)
Comorbidities (descriptive)	HTN	None	Lung adenoCA (resected) HTN	Reflux Renal cyst	None	None	Osteop.	None
Treatment characteristics								
Chemoradiotherapy regimen	Cisplatin	Cisplatin	Carbo 5-FU	Carbo 5-FU	Carbo 5-FU	Carbo 5-FU	Carbo 5-FU	Carbo 5-FU
Number of 5-FU cycles completed	N/A	N/A	3	2	2	2	3	3
Treatment toxicities and medical interventions								
Grade ≥3 toxicities (descriptive)	No	No	Mucositis Thromb.	Mucositis Dysphagia PLP Xerost.	Mucositis Dysphagia PLP Neutrop.	Mucositis Dysphagia PLP	Mucositis Aspiration Cellulitis	Mucositis Dysphagia

				Aspiration	Feb. neut.			
Enteral feeding	No	No	No	Yes	Yes	No	No	Yes
Other treatment (descriptive)	No	No	No	No	Antibiotics<br>G-CSF	No	Antibiotics	No
Hospitalization	No	No	No	No	Yes	No	Yes	No
Duration (days)	N/A	N/A	N/A	N/A	5	N/A	4	N/A

Abbreviations

HTN: hypertension
PLP: pharyngolaryngeal pain
Neutrop: neutropenia
G-CSF: growth colony stimulating factor
Thromb: thrombocytopenia
Xerost: xerostomia
Feb. neut.: febrile neutropenia
Osteop.: osteoporosis
